# Swallowing ability, nutritional status, and functioning in adults with advanced cancer excluding head, neck, and upper gastrointestinal tract: a cross-sectional study in an outpatient palliative care setting

**DOI:** 10.1590/2317-1782/e20240210en

**Published:** 2025-08-04

**Authors:** Danielle Nunes Moura Silva, Yohane Cristina Guimarães Jardim, Laélia Cristina Caseiro Vicente, Amélia Augusta de Lima Friche

**Affiliations:** 1 Programa de Pós-graduação em Ciências Fonoaudiológicas, Departamento de Fonoaudiologia, Universidade Federal de Minas Gerais – UFMG - Belo Horizonte (MG), Brasil.; 2 Oncoclínicas/MedSir - Belo Horizonte (MG), Brasil.

**Keywords:** Deglutition Disorders, Neoplasms, Health Status, Nutritional Status, Palliative Care

## Abstract

**Purpose:**

This study sought to analyze the swallowing function of cancer patients undergoing palliative care according to the degree of functionality and nutritional status.

**Methods:**

observational, cross-sectional study, conducted with advanced cancer patients outside the head and neck and upper gastrointestinal tract, in an outpatient palliative care setting at a Brazilian oncology center, conducted between March 2022 and August 2023. In the first stage, sociodemographic, clinical, functional, and nutritional data were collected. Subsequently, a speech therapy assessment was performed to classify swallowing disorders and feeding route associated with swallowing ability. Descriptive, univariate, and multivariate analyses were conducted using logistic regression.

**Results:**

39 individuals participated in the study, the majority were female, with functional performance between fair and good, moderately undernourished. Regarding the assessment of swallowing abilities, the sample mostly exhibited fully functional swallowing, followed by functional swallowing with occasional minimal cues, additional time, or avoidance of specific foods. As for the classification of swallowing, most participants had either normal swallowing or functional swallowing, with only one patient presenting mild oropharyngeal dysphagia. Multivariate analysis revealed a significant association between a decline in swallowing ability and poorer functional performance and nutritional status.

**Conclusion:**

Poorer swallowing ability in patients with advanced cancer, excluding those with head, neck, and upper gastrointestinal tract cancers, was associated with lower global functionality and nutritional status.

## INTRODUCTION

Cancer is an extremely complex condition, with an alarming incidence and a constant increase in morbidity and mortality rates, leading to a significant rise in the number of people experiencing functional dependence and longer care^(1,2)^. Palliative care (PC) is an essential form of attention at all levels of healthcare, from the diagnosis of a life-threatening disease to managing family members' grief, guided precisely by the following PC definition updated in 2019 by the World Health Organization (WHO): “An approach that improves the quality of life of patients (adults and children) and their families who are facing problems associated with life-threatening illness. It prevents and relieves suffering through the early identification, correct assessment and treatment of pain and other problems, whether physical, psychosocial or spiritual”^(3)^.

It is important to emphasize that cancer and its treatment often lead to significant health deterioration. Dysphagia, one of these consequences, is a complex, multifactorial symptom that can impact several stages of the swallowing process, including the oral, pharyngeal, and esophageal phases. This condition can lead to aspiration, malnutrition, dehydration, a considerable financial burden, and a notable reduction in quality of life (QOL) and survival^(4)^.

Worsened swallowing function has been studied little in cancer patients outside of those with head, neck, and upper gastrointestinal cancers. This degradation can manifest in patients with various types of cancer, being more common in those affecting the head and neck and the central nervous system. However, swallowing changes are also observed in cases of lymphomas and lung tumors^(5-7)^. These changes can occur across all types of cancer as an adverse effect of treatments, even when used for palliative purposes, such as surgery, radiotherapy, and chemotherapy^(5,6)^. All these contexts can impact the nutrition and quality of life of oncology patients^(8)^, especially those without prospects for cure^(9)^. The literature is still limited regarding the possible relationship between worsened swallowing ability as cancer progresses, including in the palliative phase^(9,10)^. It is also important to optimize the management of general functioning, incorporating swallowing ability and adequate, proportional nutritional support^(11)^. Thus, this study aimed to analyze the swallowing function of cancer patients in PC based on their degree of functioning and nutritional status.

## METHODS

This analytical, cross-sectional, observational study with a convenience sample was approved by the Research Ethics Committee of the Federal University of Minas Gerais, Brazil, under approval number 4,999,647.

### Participants

The study was conducted between March 2022 and August 2023, with the outpatient PC team integrated into a private oncology clinic in a capital city in Southeastern Brazil. Patients undergoing PC in this service receive individualized, simultaneous, transdisciplinary care by professionals from different areas. When necessary, care is provided separately, specifically, and exclusively by specialists in the following areas: palliative medicine, nursing, psychology, nutrition, physiotherapy, speech-language-hearing (SLH) therapy, social assistance, and pharmacy. Data were collected from patients who agreed to participate and who signed (or whose guardian signed) an informed consent form. Initially, data were collected from medical records, followed by a medical history survey and SLH assessment.

Exclusion criteria – confirmed in the medical history survey and medical records – were subjects under 18 years old; diagnosed with incurable cancer including head and neck and upper gastrointestinal tract (esophagus and stomach), classified by the Palliative Performance Scale (PPS) as equal to or below 20%; previous or recent diagnosis of traumatic brain injury, stroke, neurodegenerative, or neuromuscular diseases, facial paralysis, or craniofacial deformities; and metastases in the central nervous system.

### Access to medical records: Collection instruments and procedures

The following individual information was collected from electronic medical records: sociodemographic data (age and sex), clinical data (underlying disease, types of oncological treatments to which the patient was submitted before and during the collection period, and record of presence and intensity of symptoms), functional data (considering the following functional dimensions: ability to walk, physical performance, external evidence of disease, self-care, oral intake, state of consciousness, presence of dyspnea, edema, and delirium), and nutritional data (body measure records taken in the last consultation with the PC team and results of a validated nutritional questionnaire).

All these parameters and instruments are routinely recorded by the clinic's healthcare team, previously and constantly trained. Medical record data were accessed to collect the most recent information available, as detailed below:

- Edmonton Symptom Assessment Scale (ESAS-r), translated and validated into Portuguese^(12)^, which assesses the presence of symptoms such as pain, fatigue, drowsiness, nausea, appetite, shortness of breath, depression, anxiety, well-being, and others named by them, through visual and numerical indicators ranging from 0 to 10, with 0 being the absence of the symptom and 10 being the symptom at its greatest intensity. It is filled out by the patient and/or their caregiver in the first consultation with the PC team and reapplied whenever necessary.
- Eastern Cooperative Oncology Group Performance Status (ECOG-PS)^(13)^, which establishes scores for the functioning of oncology patients from 0 to 5, namely: 0, normal activity; 1, restricted strenuous activity; 2, more than 50% of waking hours; 3, confined to bed or chair more than 50% of waking hours; 4, 100% bedridden; and 5, dead. It is recorded by the oncologist and/or PC physician at each consultation.
- Palliative Prognostic Index (PPI)^(14,15)^, whose version translated into Brazilian Portuguese has finished the initial validation process^(15)^. Outpatients are characterized according to the PPS score, presence of delirium, dyspnea at rest, edema, and level of oral intake. The summed score categorizes the patient into one of the following three groups: Group A (PPI < 4), Group B (4 ≤ PPI ≤ 6), and Group C (PPI > 6), with predicted survival greater than 6 weeks, from 3 to 6 weeks, and less than 3 weeks, respectively^(14-17)^. This record is made by the PC physician at each consultation with the PC team.
- Palliative Performance Scale (PPS), with a free translation authorized by the Victoria Hospice Society, entitled Palliative Care Performance Scale, version 2 (PCPS v2)^(18)^. It assesses five items: ambulation, activity and evidence of illness, self-care, oral intake, and level of consciousness. Performances are divided into intervals of 10, and scores range from 100% (maximum) to 10% (minimum), with 0 being equivalent to the person’s death. It is recorded by the PC physician at each consultation.
- Body Mass Index (BMI), using the following cutoff points: thinness or underweight (BMI < 22 kg/m^2^), normal weight (BMI 22 to < 27 kg/m^2^), overweight (BMI 27 to < 30 kg/m^2^), and obesity (BMI ≥ 30 kg/m^2^), according to the Nutrition Screening Initiative – NSI 2000 criteria^(19)^. It is recorded by a nursing technician during the patient’s each in-person visit to the clinic.
- Calf circumference, using a tape measure and following the measurement method recommended by the 2018 consensus definition of sarcopenia^(20)^. It is performed by any professional during consultation with the PC team and recorded by the PC physician.
- Right and left handgrip strength ​​expressed in kilogram-force (kgf)^(21)^, measured using a Jamar^®^ hydraulic hand dynamometer (Lafayette Instrument Company, USA). It is performed by any professional during consultation with the PC team and recorded by the PC physician.
- Patient-Generated Subjective Global Assessment (PG-SGA), a validated instrument^(22)^ with a published cross-cultural adaptation in Brazilian Portuguese^(23)^. Its questionnaire classifies nutritional status into three levels (well-nourished; moderately malnourished or suspected malnutrition; and severely malnourished), and its score indicates adequate nutritional therapy: from 0 to 1, there is no need for intervention at the moment; from 2 to 3, the patient and their family members should be educated by a nutritionist or other health professional, with a need for pharmacological intervention according to the symptoms identified by the PG-SGA; from 4 to 8, reveals the need for nutritional intervention; 9 or more, critical need for improvement in symptom management and/or nutritional intervention options. It is applied by nutritionists to all PC team patients and reapplied when necessary.

### Clinical evaluation: Instruments and procedures

The clinical SLH assessment lasted an average of 30 minutes, individually, carried out by one of the two researchers working in the service, previously trained and aligned regarding the collection procedures (see Supplementary Material). They applied the following instruments: (1) SLH history survey to investigate with the patient or their companion the type and quantity of food they usually ingested; dietary restrictions; any type of adaptation in the preparation, form of presentation, or way of swallowing food and liquids; current route of nutrition/hydration; respiratory conditions; episodes of pneumonia; data on SLH therapy (if applicable); and more detailed assessment of socioeconomic levels through the Brazilian Economic Classification Criteria Questionnaire (CCEB), which classifies social class by summing points of household items and householder’s education level, with a total score ranging from 0 to 100; the higher the score, the higher the socioeconomic level^(24)^. It was carried out with the participant or companion in the first meeting.

Lastly, (2) the SLH Dysphagia Risk Evaluation Protocol (PARD, in Portuguese)^(25)^ for the clinical assessment of swallowing. This is a Brazilian protocol for classifying dysphagia, based on seven levels that include normal swallowing, functional swallowing, and five levels of oropharyngeal dysphagia. The characterization used food offered in three different consistencies, classified according to the international standardization that describes the consistencies of foods and liquids called the International Dysphagia Diet Standardization Initiative (IDDSI)^(26)^, namely: 15 ml of thin liquid (IDDSI 0), gradually offering 1 to 5 ml of filtered water at room temperature in a 5 ml syringe; 18 ml of moderately thick liquid (IDDSI 3), prepared by adding two measuring spoons, 2.4 g, of Resource ThickenUp Clear Nestlé^®^ thickener (Nestlé Health Science Company, Brazil), to 100 ml of water at room temperature, placed on a tablespoon with a 10 ml syringe, and served gradually from 3.5 ml to 10 ml; the patient was instructed to take the thickened water from the spoon and swallow each of the three fractions offered. It is important to emphasize that the evaluations with the two consistencies were not repeated three times for each volume gradation, as recommended in the original protocol, due to the frequent adverse reactions to cancer treatment, such as nausea and vomiting, in the study population. Therefore, it was decided to use a single evaluation, without repetitions, to avoid discomfort and risk to the patient. Also, Aymoré^®^ water crackers (Arcos Company, Brazil) were used to evaluate solid food intake (IDDSI 7). A stethoscope and a pulse oximeter were provided during the clinical evaluation of swallowing, as instructed by the PARD authors^(25)^. A Littmann^®^ Classic II™ pediatric stethoscope (3M Company, Brazil) and a G-Tech^®^ oximeter (Accumed-Glicomed Company, Brazil), both properly calibrated, were used to perform, respectively, cervical auscultation of swallowing sounds in the pharyngeal phase and assessment of oxygen saturation.

The final classification of swallowing ability was based on the Functional Oral Intake Scale (FOIS)^(27)^, freely translated into Portuguese, which subjectively presents scores from 1 to 7, with worse severity in the initial scores and better swallowing function in the highest score. The parameterization of the items that configured the swallowing ability was supported by the items evaluated by the PARD, as performed likewise by other authors^(28)^.

A pilot study was carried out, obtaining interrater agreement for the scales above, ranging from substantial to excellent (Kappa 0.71 to 1.00)^(29)^.

### Statistical analysis

All collected data were recorded and managed using the REDCap electronic data capture tool, a software designed to support data capture for research^(30,31)^. Statistical analyses were then performed using SPSS, version 18.0. The significance level was set at 5%. Participants' characteristics were analyzed using descriptive statistics. The response variable was swallowing functioning according to FOIS, and the explanatory variables were sociodemographic, clinical, functional, nutritional, and dietary data. Frequency distribution analysis was performed for categorical variables, and analysis of central tendency and dispersion measures for continuous variables.

To assess the association between variables, the chi-square and Fisher's exact tests were used for categorical variables and the Mann-Whitney and Kruskal-Wallis tests for quantitative variables. Univariate logistic regressions were performed to verify the factors that influenced swallowing ability. Then, multivariate analysis was performed using multiple logistic regression, selecting variables with a significance level of 25% using the backward method.

## RESULTS

The study evaluated 39, with a median age of 74 years (SD = 17.36), mostly females (69.2%). The main oncological groups according to the patients’ underlying diseases were lower gastrointestinal (41.0%), followed by breast (20.5%) and genitourinary (12.8%), all in stage IV. The most prevalent type of oncological treatment was chemotherapy (68.0%); in some cases, chemotherapy was combined with other treatments such as surgery (18.0%) and radiotherapy (7.0%).

Regarding the socioeconomic characterization of the study population, according to the CCEB, the largest concentration of the sample was in Class B2 (33.0%), followed by C1 (20.0%). The sample had a functional performance most frequently between one and two on the ECOG-PS, and more than half of the patients (59.0%) had a PPS of up to 60 (Table 1). Regarding the evidence of symptomatic particularities through ESAS-r, anxiety was the most prevalent self-reported symptom, followed by pain.

**Table 1:** Altered parameters of swallowing ability

	**1 ml**	**2 ml**	**3 ml**	**4 ml**	**5 ml**	**3 ml**	**5 ml**	**10 ml** *	**1^st^ piece**	**2^nd^ piece** **
**Extraoral spillage**										
Absent	37 (97.4%)	37 (97.4%)	36 (94.7%)	37 (97.4%)	37 (97.4%)	38 (100%)	38 (100%)	36 (100%)	36 (94.7%)	37 (100%)
Present	1 (2.6%)	1 (2.6%)	2 (5.3%)	1 (2.6%)	1 (2.6%)	0 (0%)	0 (0%)	0 (0%)	2 (5.3%)	0 (0%)
										
**Oral transit time**										
Adequate	38 (100%)	38 (100%)	38 (100%)	38 (100%)	38 (100%)	38 (100%)	38 (100%)	36 (100%)	36 (94.7%)	35 (97.4%)
Slow	0 (0%)	0 (0%)	0 (0%)	0 (0%)	0 (0%)	0 (0%)	0 (0%)	0 (0%)	2 (5.3%)	2 (5.3%)
										
**Oral cavity residue**										
Absent	38 (100%)	38 (100%)	38 (100%)	38 (100%)	38 (100%)	38 (100%)	38 (100%)	36 (100%)	37 (97.4%)	37 (100%)
Present	0 (0%)	0 (0%)	0 (0%)	0 (0%)	0 (0%)	0 (0%)	0 (0%)	0 (0%)	1 (2.6%)	0 (0%)
										
**Number of swallows per bolus**										
One	32 (84.2%)	27 (71.1%)	28 (73.7%)	27 (71.1%)	27 (71.1%)	31 (81.6%)	27 (71.1%)	24 (66.7%)	35 (92.1%)	35 (89.5%)
Multiple	6 (15.8%)	11 (28.9%)	10 (26.3%)	11 (28.9%)	11 (28.9%)	7 (18.4%)	11 (28.9%)	12 (33.3%)	3 (7.9%)	2 (5.3%)
Absent	0 (0%)	0 (0%)	0 (0%)	0 (0%)	0 (0%)	0 (0%)	0 (0%)	0 (0%)	0 (0%)	0 (0%)
										
**Laryngeal elevation**										
Adequate	36 (94.7%)	37 (97.4%)	37 (97.4%)	37 (97.4%)	37 (97.4%)	37 (97.4%)	37 (97.4%)	35 (97.4%)	37 (97.4%)	36 (97.4%)
Reduced	2 (5.3%)	1 (2.6%)	1 (2.6%)	1 (2.6%)	1 (2.6%)	1 (2.6%)	1 (2.6%)	1 (2.6%)	1 (2.6%)	1 (2.6%)
										
**Nasal reflux**										
Absent	38 (100%)	37 (97.4%)	38 (100%)	38 (100%)	38 (100%)	38 (100%)	38 (100%)	36 (100%)	38 (100%)	37 (100%)
Present	0 (0%)	1 (2.6%)	0 (0%)	0 (0%)	0 (0%)	0 (0%)	0 (0%)	0 (0%)	0 (0%)	0 (0%)
										
**Cervical auscultation**										
Adequate	38 (100%)	37 (97.4%)	38 (100%)	37 (97.4%)	37 (97.4%)	38 (100%)	37 (97.4%)	36 (100%)	38 (100%)	37 (100%)
Abnormal before and after swallowing	0 (0%)	1 (2.6%)	0 (0%)	0 (0%)	0 (0%)	0 (0%)	0 (0%)	0 (0%)	0 (0%)	0 (0%)
Abnormal after swallowing	0 (0%)	0 (0%)	0 (0%)	1 (2.6%)	1 (2.6%)	0 (0%)	1 (2.6%)	0 (0%)	0 (0%)	0 (0%)
										
**Wet voice (spontaneous laryngeal clearing)**										
Absent	38 (100%)	36 (94.7%)	38 (100%)	37 (97.4%)	37 (97.4%)	37 (97.4%)	37 (97.4%)	35 (97.4%)	38 (100%)	37 (100%)
Present	0 (0%)	2 (5.3%)	0 (0%)	1 (2.6%)	1 (2.6%)	1 (2.6%)	1 (2.6%)	1 (2.6%)	0 (0%)	0 (0%)
										
**Wet voice (voluntary laryngeal whitening)**										
Absent	38 (100%)	38 (100%)	38 (100%)	38 (100%)	37 (97.4%)	38 (100%)	37 (97.4%)	35 (97.4%)	38 (100%)	37 (100%)
Present	0 (0%)	0 (0%)	0 (0%)	0 (0%)	1 (2.6%)	0 (0%)	1 (2.6%)	1 (2.6%)	0 (0%)	0 (0%)
										
**Coughing**										
Absent	38 (100%)	36 (94.7%)	36 (94.7%)	36 (94.7%)	37 (97.4%)	38 (100%)	38 (100%)	36 (100%)	37 (97.4%)	37 (100%)
Present	0 (0%)	2 (5.3%)	2 (5.3%)	2 (5.3%)	1 (2.6%)	0 (0%)	0 (0%)	0 (0%)	1 (2.6%)	0 (0%)
										
**Cough type**										
Strong	0 (0%)	2 (5.3%)	2 (5.3%)	2 (5.3%)	1 (2.6%)	0 (0%)	0 (0%)	0 (0%)	1 (2.6%)	0 (0%)
Weak	0 (0%)	0 (0%)	0 (0%)	0 (0%)	0 (0%)	0 (0%)	0 (0%)	0 (0%)	0 (0%)	0 (0%)
Not applicable	38 (100%)	36 (94.7%)	36 (94.7%)	36 (94.7%)	37 (97.4%)	38 (100%)	38 (100%)	36 (100%)	37 (97.4%)	37 (100%)
										
**Cough mode**										
Reflex	0 (0%)	1 (2.6%)	1 (2.6%)	1 (2.6%)	0 (0%)	0 (0%)	0 (0%)	0 (0%)	0 (0%)	0 (0%)
Voluntary	0 (0%)	1 (2.6%)	1 (2.6%)	1 (2.6%)	1 (2.6%)	0 (0%)	0 (0%)	0 (0%)	1 (2.6%)	0 (0%)
Not applicable	38 (100%)	36 (94.7%)	36 (94.7%)	36 (94.7%)	37 (97.4%)	38 (100%)	38 (100%)	36 (100%)	37 (97.4%)	37 (100%)
										
**Moment of cough**										
Before	0 (0%)	0 (0%)	0 (0%)	0 (0%)	0 (0%)	0 (0%)	0 (0%)	0 (0%)	38 (100%)	0 (0%)
During	0 (0%)	2 (5.3%)	2 (5.3%)	2 (5.3%)	0 (0%)	0 (0%)	0 (0%)	0 (0%)	0 (0%)	0 (0%)
After	0 (0%)	0 (0%)	0 (0%)	0 (0%)	1 (2.6%)	0 (0%)	0 (0%)	0 (0%)	1 (2.6%)	0 (0%)
Not applicable	38 (100%)	36 (94.7%)	36 (94.7%)	36 (94.7%)	37 (97.4%)	38 (100%)	38 (100%)	36 (100%)	37 (97.4%)	37 (100%)
										
**Choking**										
Absent	38 (100%)	36 (94.7%)	38 (100%)	38 (100%)	38 (100%)	38 (100%)	38 (100%)	36 (100%)	38 (100%)	37 (100%)
Present: quick recovery	0 (0%)	2 (5.3%)	0 (0%)	0 (0%)	0 (0%)	0 (0%)	0 (0%)	0 (0%)	0 (0%)	0 (0%)
Present: difficult recovery	0 (0%)	0 (0%)	0 (0%)	0 (0%)	0 (0%)	0 (0%)	0 (0%)	0 (0%)	0 (0%)	0 (0%)
										
**Change in heart rate**										
Absent	38 (100%)	38 (100%)	38 (100%)	38 (100%)	37 (97.4%)	38 (100%)	38 (100%)	36 (100%)	37 (97.4%)	37 (100%)
Present	0 (0%)	0 (0%)	0 (0%)	0 (0%)	1 (2.6%)	0 (0%)	0 (0%)	0 (0%)	1 (2.6%)	0 (0%)
										
**Change in respiratory rate**										
Absent	38 (100%)	37 (97.4%)	36 (94.7%)	36 (94.7%)	35 (92.1%)	38 (100%)	38 (100%)	36 (100%)	37 (97.4%)	36 (97.4%)
Present	0 (0%)	1 (2.6%)	2 (5.3%)	2 (5.3%)	3 (7.9%)	0 (0%)	0 (0%)	0 (0%)	1 (2.6%)	1 (2.6%)

* 2 missing;

** 1 missing

**Figure 1:**
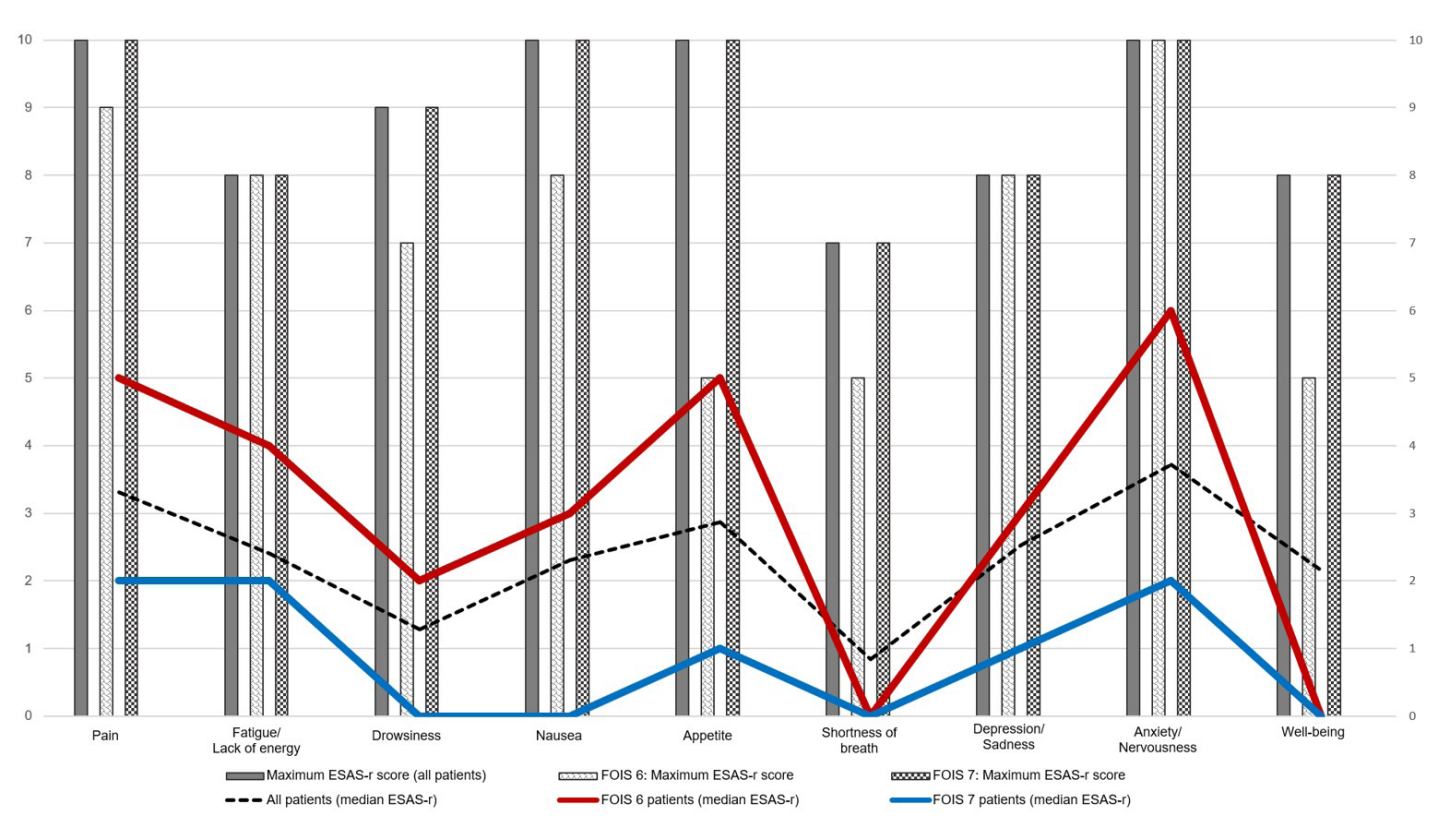
ESAS-r and swallowing

The symptoms assessed by the ESAS-r did not present statistical differences when compared by swallowing ability. However, patients with worse swallowing ability had higher medians in all symptoms assessed by the scale than those with normal swallowing and the total sample (Figure 1).

**Figure 2:**
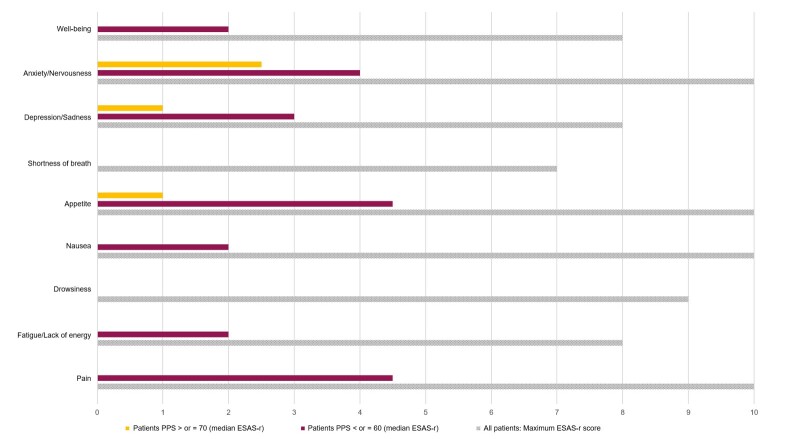
ESAS-r and PPS

On the other hand, the analysis of medians of symptoms evaluated by degree of functioning showed that pain was statistically significantly different between functioning groups (p = 0.03), as detailed in Figure 2.

The nutritional status was evidenced by the following parameters: mean BMI of 24 kg/m^2^ [±5.35], mean PG-SGA of 6 [±3.63], characterized as moderately malnourished, and the following body and strength averages: right calf circumference of 34.0 cm [±5.52], left calf circumference of 33.6 cm [±5.32], right handgrip strength of 20.0 Kgf [±7.67], and left handgrip strength of 20.2Kgf [±9.31].

The patients reported some signs and symptoms associated with swallowing, namely: xerostomia (10%), fatigue (15%), and change in taste (8%). The PARD also found slight changes in some swallowing dynamic parameters, detailed in Table 1.

Regarding swallowing skills, 74.4% had a FOIS score of 7 (considered the best functional swallowing standard), and 25.6% had a FOIS score of 6 (mild changes in the ability to ingest food and liquids). No other FOIS scores were found in the present sample. In the PARD swallowing classification, most patients had normal swallowing (63.2%), while 34.2% had functional swallowing, due to mild changes that did not impact swallowing efficiency; only one patient had mild oropharyngeal dysphagia (2.6%) due to spontaneous cough and effective voluntary throat clearing combined with mild oral changes with adequate compensations.

Finally, univariate and multivariate analysis of the sample's swallowing function was performed to demonstrate probable relationships with the degree of functioning and nutritional status. The levels of swallowing ability were statistically significantly associated with ECOG-PS and PG-SGA results, as shown in Table 2.

**Table 2:** Swallowing ability according to clinical-functional and nutritional dimensions

	FOIS 6 N = 10	FOIS 7 N = 29	Odds Ratio [95%CI]	p-value
**CATEGORICAL MEASURES**	**N (%)**			
**Sex**				
Females	5 (18.5%)	22 (81.5%)	-----	0.394LR
Males	5 (41.7%)	7 (51.3%)		
**Self-reported xerostomia**				
Yes	2 (50.0%)	2 (50.0%)	-----	0.267^C^
No	8 (22.9%)	27 (77.1%)		
**Self-reported fatigue**				
Yes	1 (16.7%)	5 (83.3%)	-----	1.000^C^
No	9 (27.3%)	24 (72.7%)		
**Self-reported dysgeusia**				
Yes	1 (33.3%)	2 (66.7%)	-----	1.000^C^
No	9 (25.0%)	27 (75.0%)		
**Self-reported recent pneumonia**				
Yes	2 (66.7%)	1 (33.3%)	-----	0.256^LR^
No	8 (22.2%)	28 (77.8%)		
**Nutritional status**				
Well nourished	1 (6.7%)	14 (93.3%)	-----	0.331^LR^
Moderately to Severely Malnourished**	9 (37.5%)	15 (62.5%)		
**PPS**				
PPS < or = 60	8 (34.8%)	15 (65.2%)	-----	0.126^LR^
PPS > or = 70	2 (12.5%)	14 (87.5%)		
CONTINUOUS MEASURES	(Mean/Median[Standard deviation])		Hazard Ratio [95%CI]	
CCEB (total score)	31.50 [±8.90]	32.00 [±9.71]	-----	0.856^LR^
Age (years)	73 [±17.26]	69 [±17.04]	-----	0.518^LR^
BMI (Kg/m^2^)	25.84 [±6.26]	24.63 [±5.29]		0.489^M^
Right handgrip (kgf)+	19 [±7.79]	20 [±7.52]	-----	0.488^M^
Left handgrip (kgf)^+^	19 [±10.31]	21 [±9.70]	-----	0.459^M^
Right calf circumference (cm)++	34 [±5.52]	34 [±4.62]	-----	0.390^M^
Left calf circumference (cm) ^++^	34 [±6.46]	34 [±4.71]	-----	0.298^M^
PG-SGA	8 [±5.42]	5 [±3.01]	0.76 [0.59 - 0.98]**	0.040^LR^
ECOG-PS	2.17 [±0.75]	1.25 [±0.85]	0.23 [0.57 - 0.95]**	0.044^LR^
PPI	2.83 [±1.57]	1.28 [±1.90]	-----	0.367^LR^

C Chi-square;

M Mann-Whitney;

** OR adjusted according to FOIS multivariate analysis for age, sex, nutritional status, recent pneumonia, PPS, and PPI (backward p < 0.25);

LR p-value according to the respective last logistic regression model;

+ 5 missing;

++ 8 missing

## DISCUSSION

This study assessed swallowing function according to the degree of functioning and nutritional status of patients undergoing oncology PC, except for those who covered anatomical regions of swallowing. The main results were associations between minimal change in swallowing ability and worse levels of both global and nutritional functioning in this sample. Although the root cause of this causality is unknown, these data should be considered.

In the study sample, 25.6% had mild changes in the ability to ingest food and liquids according to FOIS, due to spontaneous and effective compensations in the oral and pharyngeal phases of swallowing. All nutrition and hydration were maintained orally. This corroborates the findings of another cross-sectional study of patients with cancer outside the anatomical regions of swallowing, which considered the presence of dysphagia if the FOIS scale was less than 7; hence, dysphagia occurred in 19%, reaching 30% of those in PC. Thus, both studies highlight a new way of considering swallowing functioning, in addition to the importance of excluding head, neck, and upper gastrointestinal tract cancers from such analyses.

On the other hand, similar results were observed in studies with patients with any type of advanced cancer, including anatomical areas of swallowing. Another cross-sectional study, carried out in an outpatient setting with patients with any type of cancer, found that 56.7% scored 7, and 23.2% scored 6 in the FOIS^(32)^. An Italian prospective cohort^(33)^ observed dysphagia in 15% of the total patients, and most of these were classified as a swallowing disorder that partially affected the patients’ nutrition, without needing oral supplementation or an alternative route. These results highlight the importance of etiologically distinguishing the anatomical and physiological changes that impact the swallowing function. In cases of head and neck cancer, including advanced ones, these changes occur due to structural muscular, bone, and cartilaginous deformations of the digestive and/or respiratory tract^(34)^. In cases of central nervous system cancer, neurological structural changes cause neurogenic dysphagia^(11)^. Hence, dysphagia resulting from oncological diseases outside the anatomical areas of swallowing appears to be explained by the clinical and functional degradation promoted by cancer and its treatments, mainly chemotherapy^(7,11)^. The impact on the loss of strength and mobility of the body's overall muscles during cancer treatment includes the muscles involved in swallowing^(11)^. This process appears to affect the biomechanical and sensory mechanisms of swallowing, as seen in the present sample, possibly contributing to the emergence of oropharyngeal or esophageal dysphagia due to decreased functioning^(11)^ – which we propose to call dysphagia due to functional decline, a gastrointestinal symptom that needs to be managed mainly in patients with advanced cancer.

The relationship between impaired swallowing ability, even if slight, and worse degree of functioning in this study’s sample also corroborates the muscular-functional impact that oncology has on the swallowing ability, as mentioned by Okuni et al.^(11)^. Furthermore, Italian researchers^(33)^ found a relationship between dysphagia and a low functioning on a scale. The researchers of two other studies in patients with advanced cancer^(10,35)^ found that dysphagia is one of the most common symptoms in the last 7 days of life and the last hours of life, respectively, which shows high clinical-functional degradation related to this gastrointestinal symptom.

The relationship between functional decline and some ESAS-r symptoms found in this study corroborates the findings of other Brazilian researchers^(36)^. They likewise found pain (mean and median of 4.04 and 5.0, respectively) and anxiety (3.85 and 4.0) as the most prevalent symptoms in a palliative outpatient setting. However, they did not analyze the relationship with functional status.

Nutritional aspects in the present sample were statistically significantly related to worse functioning and changes in swallowing ability. The relationship with functioning was also found by Oliveira et al.^(37)^ in a population of Brazilians with various types of incurable cancer treated mostly in oncology clinics, in which the best PG-SGA qualifications reflected better physical aspects and overall quality of life. However, another Brazilian cross-sectional study with cancer patients receiving only PC showed no association between functional performance according to the PPS and the nutritional aspects evaluated. This lack of association may be related to the small sample size, a justification highlighted by the authors. The national^(38,39)^ and international^(4,33,40)^ literature has highlighted the association between nutritional status and dysphagia for some time, and the findings of the present study are in line with the scientific community.

In view of the growing number of patients with advanced cancer worldwide, there is a need to strengthen public policies aimed at the PC model to compose PC teams with professionals from different areas, at all levels of healthcare, whether public or private. Moreover, health teams must be aware of the multiple care required by the complexity and uniqueness of patients with advanced cancer^(41)^. Changes in oral functions integrate this complex clinical-functional deterioration, even in regions other than the head, neck, and upper gastrointestinal tract. Therefore, they need to be better recognized and managed^(42)^. This study highlights the crucial role of swallowing for these patients’ nutrition, satisfaction, and quality of life.

There are some limitations to our study. First, it did not use instrumental assessment, such as videofluoroscopic swallowing study (VFSS), due to unfavorable geographic logistics – the collection site did not have the equipment, and patients would have to travel to another place for examination. In contrast, a protocol that evaluates more than one food consistency (PARD) was used precisely because this type of assessment has a sensitivity of 90%, compared to VFSS^(43)^, evidencing the equivalence of the complete clinical assessment, which can be considered sovereign. Second, this study may not have controlled all possible confounding factors derived from such a heterogeneous sample. However, we performed multivariate analyses, which gave greater robustness to the results. Third, the selection bias of participants should be considered. The sample was constituted by convenience due to the difficulty of recruitment, and a deteriorated health condition was one of the reasons for refusal. These barriers related to patients being monitored by a PC team were also identified by other authors^(44,45)^.

Despite these limitations, a crucial strength is that this research provides the prevalence of dysphagia in cancer patients who do not encompass anatomical areas of swallowing and its relationship with clinical-functional and nutritional degradation through primary outcome, which lends credibility to our findings and greatly contributes to improving care for these patients and for PC.

Further randomized research is needed, including multicenter studies, to deepen the understanding of symptoms and their impacts and to analyze in depth the results of the holistic approach in patients with incurable diseases and their families.

## CONCLUSION

In conclusion, this study shows that impaired swallowing ability, even though slight in this sample of patients with advanced cancer not involving the head, neck, or upper gastrointestinal tract, is related to a lower degree of functioning and deficient nutritional status.

## Supplementary Material

Supplementary material accompanies this paper.

Pilot project and interrater agreement analysis

This material is available as part of the online article from https://doi.org/10.1590/2317-1782/e20240210en
