# Supplementary material for: Swallowing ability, nutritional status, and functioning in adults with advanced cancer excluding head, neck, and upper gastrointestinal tract: a cross-sectional study in an outpatient palliative care setting
Source: Codas. 2025 Aug 4;37(4):e20240210. doi: 10.1590/2317-1782/e20240210en (PMC12547879; doi:10.1590/2317-1782/e20240210en)
Supplement: Pilot project and interrater agreement analysis [file codas-37-4-e20240210-suppl01-en.pdf]

SUPPLEMENTARY MATERIAL

Pilot project and interrater agreement analysis

The two examiners underwent prior training on the protocols and scales used in the study before starting the clinical data collection. Then, a pilot study was carried out with 10% of the sample estimated by the sample calculation to test the methodological viability of the proposed instruments and procedures, as the literature recommends[(1)]. The patients were simultaneously evaluated by the two SLH pathologists (field investigators) independently, and the intrarater and interrater agreement was performed using the Kappa coefficient[(2)]. The agreement between the two raters in each PARD topic ranged from substantial to excellent (the lowest Kappa = 0.39, and the highest = 1.00). This variation can be justified because the protocol items are based on subjective analyses influenced by theoretical training and previous clinical experiences. In contrast, this discrepancy did not influence the agreement for the final diagnostic criteria, which were exclusively excellent (PARD = 1.00; ASHA = 1.00). They held discussions, alignments, and proposed adjustments to improve the agreement on the PARD protocol topics, followed by a new pilot with 5% of the sample estimated by the sample calculation to test the calibration and adjustments for the definitive data collection. Thus, agreement ranged from substantial to excellent (the lowest Kappa = 0.71, and the highest = 1.00), maintaining excellent agreement for the final diagnostic criteria (PARD = 1.00; ASHA = 1.00).

Supplementary material references

1. Scola LFC, Moseley AM, Thabane L, Almeida M, Costa LDCM. A methodological survey on reporting of pilot and feasibility trials for physiotherapy interventions: a study protocol. BMJ Open. 2019 May;9(5):e020580. http://dx.doi.org/1010.1136/bmjopen-2017-020580

2. Landis JR, Koch GG. The measurement of observer agreement for categorical data. Biometrics. 1977 Mar;33(1):159-74. PMID: 843571.

MATERIAL SUPLEMENTAR

Projeto piloto e análise de concordância entre examinador

É importante ressaltar, que antes de iniciar a coleta clínica propriamente dita, as duas examinadoras foram submetidas a prévio treinamento dos protocolos e escalas utilizadas no estudo. Em seguida, foi realizado um estudo piloto com 10% da amostra estimada pelo cálculo amostral a fim de testar a viabilidade metodológica dos instrumentos e procedimentos propostos, como preconizado em literatura(1). Os pacientes foram simultaneamente avaliados pelas duas fonoaudiólogas investigadoras de campo de forma independente, e a concordância intra e entre as avaliadores foi realizada por meio do coeficiente Kappa(2). A concordância entre as duas avaliadoras em cada tópico do protocolo PARD transitaram entre razoável e excelente (o menor Kappa= 0,39 e o maior = 1,00). Esta variação pode ser justificada devido aos itens do protocolo supracitado se basear em análises subjetivas influenciadas por formação teórica e experiências clínicas prévias. Em contraponto, tal discrepância não influenciou na concordância para os critérios diagnósticos finais, que foram exclusivamente excelentes (PARD = 1,00 e ASHA = 1,00). A fim de melhorar a concordância dos tópicos do protocolo PARD, foram realizados discussões, alinhamentos e propostos ajustes, seguido de novo piloto com 5% da amostra estimada pelo cálculo amostral a fim de testar a calibração e adequações para a coleta de dados definitiva, encontrando assim concordância entre substancial e excelente (o menor Kappa= 0,71 e o maior = 1,00), E mantendo-se concordância para os critérios diagnósticos finais excelentes (PARD = 1,00 e ASHA = 1,00).

Referências material suplementar

1. Scola LFC, Moseley AM, Thabane L, Almeida M, Costa LDCM. A methodological survey on reporting of pilot and feasibility trials for physiotherapy interventions: a study protocol. BMJ Open. 2019 May;9(5):e020580. http://dx.doi.org/1010.1136/bmjopen-2017-020580

2. Landis JR, Koch GG. The measurement of observer agreement for categorical data. Biometrics. 1977 Mar;33(1):159-74. PMID: 843571.
