# Supplementary material for: Swallowing ability, nutritional status, and functioning in adults with advanced cancer excluding head, neck, and upper gastrointestinal tract: a cross-sectional study in an outpatient palliative care setting
Source: Codas. 2025 Aug 4;37(4):e20240210. doi: 10.1590/2317-1782/e20240210en (PMC12547879; doi:10.1590/2317-1782/e20240210en)
Supplement: Translation

# Translation: Habilidade de deglutição, estado nutricional e funcionalidade de adultos com câncer avançado exceto cabeça, pescoço e trato gastrointestinal superior: um estudo transversal em um ambulatório de cuidados paliativos

**DOI:** 10.1590/2317-1782/e20240210pt

**Authors:** Danielle Nunes Moura Silva, Yohane Cristina Guimarães Jardim, Laélia Cristina Caseiro Vicente, Amélia Augusta de Lima Friche

**Affiliations:** 1 Programa de Pós-graduação em Ciências Fonoaudiológicas, Departamento de Fonoaudiologia, Universidade Federal de Minas Gerais – UFMG - Belo Horizonte (MG), Brasil.; 2 Oncoclínicas/MedSir - Belo Horizonte (MG), Brasil.

**Keywords:** Transtornos de Deglutição, Neoplasias, Nível de Saúde, Estado Nutricional, Cuidados Paliativos

## Abstract

**Objetivo:**

Analisar a função de deglutição de pacientes oncológicos em cuidados paliativos segundo a funcionalidade e o estado nutricional.

**Método:**

Estudo observacional, do tipo transversal, com pacientes com câncer avançado excluindo cabeça, pescoço e trato gastrointestinal superior, em cuidados paliativos ambulatorial de um centro oncológico brasileiro, realizado entre março de 2022 e agosto de 2023. Foram obtidos dados sociodemográficos, clínicos, funcionais e nutricionais. A avaliação fonoaudiológica classificou a habilidade de deglutição e a via de alimentação por meio dos protocolos PARD e FOIS, respectivamente. Foram realizadas análise descritiva, uni e multivariada por meio de regressão logística.

**Resultados:**

39 indivíduos participaram do estudo, a maioria do sexo feminino, performance funcional entre regular a boa e moderadamente desnutridos. Na avaliação das habilidades de deglutição, os pacientes, majoritariamente, foram classificados com deglutição plenamente funcional, seguida de deglutição funcional com necessidades raras de mínimas pistas, tempo adicional ou evitar alimentos específicos. Quanto à classificação da deglutição, a maioria dos participantes apresentou deglutição normal ou deglutição funcional e apenas um paciente apresentou disfagia orofaríngea leve. A análise multivariada revelou associação significativa entre pior habilidade de deglutição e desempenho funcional e estado nutricional inferiores.

**Conclusão:**

Habilidade de deglutição prejudicada em pacientes com câncer avançado que não abarcam a região de cabeça, pescoço e trato gastrointestinal superior foi associada a uma funcionalidade global e estado nutricional inferiores.

## INTRODUÇÃO

O câncer se caracteriza por uma condição extremamente complexa, com incidência alarmante e constante ascensão da taxa de morbidade e mortalidade, o que resulta em um aumento significativo do número de pessoas que vivenciam quadros de dependência funcional e do tempo que necessitarão de cuidados^(1,2)^. Os cuidados paliativos (CP) são uma modalidade de cuidado indispensável em todos os níveis assistenciais de saúde desde o diagnóstico de doença ameaçadora a vida até o manejo do luto dos familiares, pautada justamente pela definição atualizada em 2019 pela Organização Mundial de Saúde (OMS) que define: “uma abordagem de melhora da qualidade de vida dos pacientes (adultos ou crianças) e de seus familiares, que enfrentam problemas associados a doenças que ameaçam a vida. Previne e alivia sofrimento por meio da investigação precoce, avaliação correta e tratamento da dor e de outros problemas físicos, psicossociais ou espirituais”.^(3)^

É importante destacar que o câncer e seu tratamento frequentemente desencadeiam deterioração significativas da saúde, sendo a disfagia uma delas. A disfagia é um sintoma complexo e multifatorial que pode afetar várias etapas do processo de deglutição, incluindo as fases oral, faríngea e esofágica, com consequências como aspiração, desnutrição, desidratação, encargos financeiros significativos, bem como em uma redução substancial da qualidade de vida (QV) e da sobrevida^(4)^.

A piora da funcionalidade da deglutição é pouco estudada em pacientes com câncer fora da região de cabeça e pescoço e sistema gastrointestinal alto. Na população de pacientes oncológicos, essa degradação pode se manifestar em paciente com diversos tipos de câncer, sendo mais comuns naquelas que acometem a região de cabeça e pescoço, bem como o sistema nervoso central. Entretanto, as alterações de deglutição podem também ser observadas em casos de linfomas e tumores de pulmão^(5-7)^. Além disso, surgem como efeito adverso dos tratamentos empregados, mesmo quando utilizados com finalidades paliativas, em todas as modalidades de câncer, tais como cirurgia, radioterapia e quimioterapia^(5,6)^. Todos esses contextos podem ter impacto na nutrição e na qualidade de vida dos pacientes oncológicos^(8)^ e principalmente daqueles que não têm perspectivas de cura^(9)^. A literatura ainda é incipiente quanto a possível relação da piora da habilidade de deglutição à medida que o câncer progride, incluindo a fase paliativa^(9,10)^. Ressalta-se ainda a importância do manejo otimizado da funcionalidade geral, incluindo a habilidade de deglutição e a oferta de suporte nutricional adequado e proporcional^(11)^. Diante do exposto, o presente estudo tem como objetivo analisar a função de deglutição de pacientes oncológicos em cuidados paliativos segundo o grau de funcionalidade e o estado nutricional.

## MÉTODO

Trata-se de um estudo observacional analítico, do tipo transversal, composto por amostra de conveniência. Este trabalho foi aprovado pelo Comitê de Ética em Pesquisa da Universidade Federal de Minas Gerais (COEP), Brasil, sob o parecer 4.999.647.

### Participantes

Estudo conduzido entre março de 2022 e agosto de 2023, junto à equipe CP ambulatorial integrada a uma clínica oncológica privada de uma capital do sudeste brasileiro. Neste serviço, os pacientes em cuidados paliativos recebem atendimento individualizado de forma simultânea e transdisciplinar por profissionais de diversas áreas. Quando necessário, o atendimento é realizado em separado de forma específica e exclusiva por especialistas das seguintes áreas: medicina paliativa, enfermagem, psicologia, nutrição, fisioterapia, fonoaudiologia, assistência social e farmácia. A coleta de dados aconteceu com os pacientes que concordaram em participar e que assinaram (ou o responsável legal) o Termo de Consentimento Livre e Esclarecido (TCLE). Inicialmente, foi realizada coleta de dados dos prontuários e em seguida se executou anamnese e avaliação fonoaudiológica.

Foram critérios de exclusão - confirmadas na anamnese e por acesso a prontuário - sujeitos com idade inferior a 18 anos; diagnosticados com câncer incurável que abarcavam os sítios de cabeça e pescoço e de trato gastrointestinal superior (esôfago e estômago), classificados pela escala de performance paliativa, a *Palliative Performance Status* (PPS), igual ou abaixo de 20%; diagnóstico prévio ou recente de traumatismo crânio encefálico, acidente vascular encefálico, doenças neurodegenerativas e neuromusculares, paralisia facial ou deformidades craniofaciais, e metástases em sistema nervoso central.

### Acesso a prontuários: instrumentos e procedimentos de coleta

As seguintes informações individuais foram coletadas em prontuário eletrônico: dados sociodemográficos: idade e sexo; dados clínicos: doença de base, tipos de tratamentos oncológicos aos quais o paciente foi submetido antes e durante o período de coleta, bem como a investigação do registro de presença e intensidade de sintomas; dados funcionais, considerando as seguintes dimensões funcionais: capacidade de deambular, performance física, evidência exterior de doença, autocuidado, ingesta oral, estado de consciência, presença de dispneia, edema e delirium; e finalmente dados nutricionais, mediante anotação das medidas corporais em última consulta com equipe CP e resultados de questionário nutricional validado.

Todos esses parâmetros e instrumentos são rotineiramente registrados pela equipe assistencial da clínica, previamente e constantemente treinada. Estes registros em prontuário foram acessados visando coletar as informações mais recentes disponíveis, e seguem detalhados a seguir:

- Escala de Avaliação de Sintomas de *Edmonton* (ESAS-r), traduzida e validada para o português^(12)^, que avalia a presença de sintomas como dor, cansaço, sonolência, náusea, apetite, falta de ar, depressão, ansiedade, bem-estar, e outros que forem nomeados, por meio de indicadores visuais e numéricos que variam entre 0 e 10, sendo 0 a ausência do sintoma e 10 o sintoma em sua maior intensidade, preenchida pelo paciente e/ou seu cuidador na primeira consulta com a equipe CP e reaplicada sempre que necessário;
- Escala de capacidade funcional *Eastern Cooperative Oncology Group Performance Status* (ECOG-PS)^(13)^ que estabelece escores da funcionalidade de pacientes oncológicos de 0 a 5, a saber: 0, atividade normal; 1, atividade extenuante restrita; 2, mais de 50% das horas em vigília; 3, confinado à cama ou cadeira mais que 50% das horas de vigília; 4, 100% acamado; e 5, morto. Registrada pelo médico oncologista e/ou paliativista a cada consulta;
- Escore *Palliative Prognostic Index* (PPI)^(14,15)^, conta também com uma versão traduzida para português brasileiro em processo de validação inicial finalizado^(15)^. Para o cenário ambulatorial, caracterizam o paciente segundo o escore PPS, presença de delirium, dispneia em repouso, edema e nível de ingestão oral, onde a soma dos escores produz a categorização do paciente em um dos três seguintes grupos: Grupo A (PPI <4), Grupo B (4≤PPI≤6) e Grupo C (PPI>6) com sobrevida predita maior que seis semanas, entre três e seis semanas e menor que três semanas, respectivamente^(14-17)^. Esse registro é realizado pelo médico paliativista a cada consulta com a equipe CP;
- *Palliative Performance Scale* (PPS), com tradução livre autorizada pela Victoria Hospice Society, intitulada Escala de Desempenho em Cuidados Paliativos versão 2 (EDCP v2)^(18)^, que avalia cinco itens: deambulação, atividade e evidência de doença, autocuidado, ingesta oral e nível de consciência. As performances são divididas em intervalos de dez, e as pontuações variam de 100% (máxima) a 10% (mínima), sendo zero equivalente à morte do indivíduo - registradas pelo médico paliativista a cada consulta;
- Índice de Massa Corporal (IMC), utilizando-se os pontos de corte: magreza ou baixo peso (IMC < 22 kg/m2); eutrofia (IMC 22 a < 27 kg/m2); sobrepeso (IMC 27 a < 30 kg/m2); e obesidade (IMC ≥ 30 kg/m2), segundo os critérios de Nutrition Screening Initiative – NSI 2000^(19)^ - registrado por técnico de enfermagem em toda visita presencial do paciente à clínica;
- Medição da circunferência da panturrilha - com fita métrica e seguindo a forma de mensuração recomendada pelo consenso de definição de sarcopenia de 2018^(20)^. Executado por qualquer profissional durante a consulta com a equipe de CP e registrado pelo médico paliativista;
- Valores de força de preensão manual (*Handgrip*) direita e esquerda expressos em quilograma força (kgf)^(21)^, mensurada por meio do dinamômetro manual hidráulico Jamar® (Lafayette Instrument Company, EUA). Executado por qualquer profissional durante a consulta com a equipe de CP e registrado pelo médico paliativista;
- Avaliação Subjetiva Global Produzida Pelo Paciente (ASG-PPP), instrumento validado^(22)^ e com publicação de adaptação transcultural em língua portuguesa do Brasil^(23)^, caracteriza-se como um questionário de classificação do estado nutricional em três níveis (Bem nutrido; Moderadamente desnutrido ou suspeita de desnutrição; e Gravemente desnutrido) e possibilita o cálculo de pontuação que indica a terapia nutricional adequada: de 0 a 1, não há necessidade de intervenção no momento; de 2 a 3, o paciente e seus familiares devem ser educados por nutricionista ou outro profissional de saúde, com necessidade de intervenção farmacológica de acordo com os sintomas identificados pela ASG-PPP; de 4 a 8, revela a necessidade de intervenção nutricional; 9 ou mais, necessidade crítica de melhora no manuseio dos sintomas e/ou opções de intervenção nutricional. Esse questionário é aplicado pelos nutricionistas em todos os pacientes da equipe CP e reaplicado quando necessário.

### Avaliação clínica: instrumentos utilizados e procedimentos realizados

A avaliação fonoaudiológica clínica ocorreu com duração média de 30 minutos, de forma individual e foi realizada por uma das duas pesquisadoras que atuam no serviço, previamente treinadas e alinhadas em relação aos procedimentos de coleta (vide Material Suplementar). Foram aplicados os seguintes instrumentos: (1) Anamnese fonoaudiológica, com a finalidade de realizar investigação com o paciente ou seu acompanhante quanto ao tipo e quantidade de alimento habitualmente ingerido pelo paciente, presença de restrições alimentares, pesquisa de algum tipo de adaptação no preparo, na forma de apresentação e no modo de deglutir os alimentos e líquidos, anotação da via atual de nutrição/hidratação, informações sobre condições respiratórias, episódios de pneumonia e dados sobre a terapia fonoaudiológica - se fosse o caso, e apuração mais detalhada dos níveis socioeconômicos por meio da aplicação do Questionário Critério de Classificação Econômica Brasil (CCEB), instrumento que possibilita classificar a classe social por meio da soma de pontos de itens no domicílio e da escolaridade do chefe da família, com variação do escore total de 0 a 100, sendo que quanto maior a pontuação maior o nível socioeconômico^(24)^ - realizado com o próprio participante ou acompanhante, no primeiro encontro.

E finalmente, (2) Protocolo Fonoaudiológico de Avaliação do Risco para Disfagia (PARD)^(25)^, para a avaliação clínica da deglutição. Trata-se de protocolo brasileiro para classificação da disfagia, baseada em sete níveis que incluem deglutição normal, deglutição funcional e cinco níveis de disfagia orofaríngea. Para essa caracterização, foram ofertados alimentos em três diferentes consistências, classificadas de acordo a padronização internacional que descreve as consistências de alimentos e líquidos intitulada *International Dysphagia Diet Standardisation Initiative* (IDDSI)^(26)^, são eles: 15 ml líquido ralo (IDDSI 0), ofertado gradativamente de 1 a 5ml de água filtrada em temperatura ambiente em seringa de 5 ml; 18ml de alimento liquidificado ou moderadamente espessado (IDDSI 3, consistência elaborada por meio da adição de duas colheres de medida, 2,4g, do espessante *Resource ThickenUp Clear* Nestlé**®** (Nestlé Health Science Company, Brasil), em 100ml de água em temperatura ambiente), colocado na colher de sobremesa por uma seringa de 10 ml, gradativamente 3,5 ml e 10 ml, sendo o paciente orientado a capturar a água espessada da colher e deglutir à oferta de cada uma das três frações. Importante ressaltar que as avaliações com as duas consistências não foram repetidas três vezes para cada gradação de volume, conforme recomendado no protocolo original, devido à frequente presença de reações adversas ao tratamento oncológico como náusea e vômito pela população estudada. Assim, a fim de eximir desconforto e risco ao paciente, optou-se pela avaliação pontual, sem repetições. Para a avaliação da ingestão de alimento sólido (IDDSI 7), foi utilizado biscoito Água e Sal Aymoré® (Arcos Company, Brasil). Conforme orientado pelos autores do PARD^(25)^, durante a avaliação clínica da deglutição foram dispostos de estetoscópio e de oxímetro de pulso. Os equipamentos estavam devidamente calibrados, sendo utilizado o estetoscópio *Littmann*® modelo *Classic II™* Pediátrico (3M Company^,^ Brasil) e oxímetro *G-Tech*® (Accumed-Glicomed Company, Brasil), para realização, respectivamente, de ausculta cervical dos ruídos da deglutição na fase faríngea e avaliação da saturação de oxigênio.

A classificação final da habilidade de deglutição se baseou pela escala *Functional Oral Intake Scale* (FOIS)^(27)^, com tradução livre para o português e que, subjetivamente, apresenta escores de 1 a 7, sendo pior gravidade nos escores iniciais e melhor função de deglutição no maior escore. A parametrização dos itens que configuraram a habilidade de deglutição foi apoiado nos itens avaliados pelo PARD, conforme também realizado por outros autores^(28)^.

Importante ressaltar que foi realizado estudo piloto e obteve-se concordância interavaliadores das escalas acima explicitadas, que variou entre substancial e excelente (Kappa 0,71 a 1,00)^(29)^.

### Análise estatística

Todos os dados coletados foram registrados e tiveram seus dados gerenciados em ferramenta de captura eletrônica de dados *REDCap*, software projetado para apoiar a captura de dados para pesquisas^(30,31)^. Em seguida, as análises estatísticas foram realizadas utilizando SPSS, versão 18.0. O nível de significância foi fixado em 5%. As características dos participantes foram analisadas por meio de estatística descritiva. A variável resposta foi a funcionalidade da deglutição segundo FOIS, e as variáveis explicativas foram os dados sociodemográficos, clínicos, funcionais, nutricionais e alimentares. Foram realizadas análise da distribuição de frequência para as variáveis categóricas e análise das medidas de tendência central e dispersão para as variáveis contínuas.

Para avaliação da associação entre as variáveis foram utilizados os testes Qui-quadrado e exato de Fisher para as variáveis categóricas e Mann-Whitney e Kruskall Wallis para as variáveis quantitativas. Regressões logísticas univariadas foram realizadas para verificação dos fatores influenciáveis na habilidade de deglutição. Na sequência, foi realizada análise multivariada, por meio de regressão logística múltipla, selecionando as variáveis com nível de significância de 25% por meio do método Backward.

## RESULTADOS

Foram avaliados 39 indivíduos, com mediana de idade de 74 anos (DP=17,36). A maioria era do sexo feminino (69,2%). Os principais grupos oncológicos como doença de base apresentadas pelos pacientes foram gastrointestinal baixo (41,0%), seguido por mama (20,5%) e geniturinário (12,8%) e todos encontravam-se no nível de estadiamento IV. O tipo de tratamento oncológico mais prevalente foi a quimioterapia (68,0%), sendo que em alguns casos, a quimioterapia foi combinada com outros tratamentos como cirurgia (18,0%) e radioterapia (7,0%).

Quanto à caracterização socioeconômica da população estudada, de acordo com o Critério de Classificação Econômica Brasil (CCEB), a maior concentração da amostra era de Classe B2 (33,0%), seguida de C1 (20,0%). A população estudada possuía performance funcional mais frequente entre um e dois na escala ECOG-PS e em mais da metade dos pacientes (59,0%) foi observado PPS de até 60 (Tabela 1). Quanto à evidência de particularidades sintomáticas por meio da escala ESAS-r, ‘Ansiedade’ foi o sintoma autorreferido mais prevalente, seguido do sintoma ‘Dor’.

**Tabela 1:** Parâmetros alterados da habilidade de deglutição

	1 ml	2 ml	3 ml	4 ml	5 ml	3 ml	5 ml	10 ml*	1º pedaço	2º pedaço**
**Escape extraoral**										
ausência	37 (97,4%)	37 (97,4%)	36 (94,7%)	37 (97,4%)	37 (97,4%)	38 (100%)	38 (100%)	36 (100%)	36 (94,7%)	37 (100%)
presença	1 (2,6%)	1 (2,6%)	2 (5,3%)	1 (2,6%)	1 (2,6%)	0 (0%)	0 (0%)	0 (0%)	2 (5,3%)	0 (0%)
										
**Tempo de trânsito oral**										
adequado	38 (100%)	38 (100%)	38 (100%)	38 (100%)	38 (100%)	38 (100%)	38 (100%)	36 (100%)	36 (94,7%)	35 (97,4%)
lento	0 (0%)	0 (0%)	0 (0%)	0 (0%)	0 (0%)	0 (0%)	0 (0%)	0 (0%)	2 (5,3%)	2 (5,3%)
										
**Resíduo em cavidade oral**										
ausência	38 (100%)	38 (100%)	38 (100%)	38 (100%)	38 (100%)	38 (100%)	38 (100%)	36 (100%)	37 (97,4%)	37 (100%)
presença	0 (0%)	0 (0%)	0 (0%)	0 (0%)	0 (0%)	0 (0%)	0 (0%)	0 (0%)	1 (2,6%)	0 (0%)
										
**Número de deglutições por bolo**										
única	32 (84,2%)	27 (71,1%)	28 (73,7%)	27 (71,1%)	27 (71,1%)	31 (81,6%)	27 (71,1%)	24 (66,7%)	35 (92,1%)	35 (89,5%)
múltiplas	6 (15,8%)	11 (28,9%)	10 (26,3%)	11 (28,9%)	11 (28,9%)	7 (18,4%)	11 (28,9%)	12 (33,3%)	3 (7,9%)	2 (5,3%)
ausente	0 (0%)	0 (0%)	0 (0%)	0 (0%)	0 (0%)	0 (0%)	0 (0%)	0 (0%)	0 (0%)	0 (0%)
										
**Elevação laríngea**										
adequada	36 (94,7%)	37 (97,4%)	37 (97,4%)	37 (97,4%)	37 (97,4%)	37 (97,4%)	37 (97,4%)	35 (97,4%)	37 (97,4%)	36 (97,4%)
reduzida	2 (5,3%)	1 (2,6%)	1 (2,6%)	1 (2,6%)	1 (2,6%)	1 (2,6%)	1 (2,6%)	1 (2,6%)	1 (2,6%)	1 (2,6%)
										
**Refluxo nasal**										
ausência	38 (100%)	37 (97,4%)	38 (100%)	38 (100%)	38 (100%)	38 (100%)	38 (100%)	36 (100%)	38 (100%)	37 (100%)
presença	0 (0%)	1 (2,6%)	0 (0%)	0 (0%)	0 (0%)	0 (0%)	0 (0%)	0 (0%)	0 (0%)	0 (0%)
										
**Ausculta cervical**										
adequada	38 (100%)	37 (97,4%)	38 (100%)	37 (97,4%)	37 (97,4%)	38 (100%)	37 (97,4%)	36 (100%)	38 (100%)	37 (100%)
alterada antes e após a deglutição	0 (0%)	1 (2,6%)	0 (0%)	0 (0%)	0 (0%)	0 (0%)	0 (0%)	0 (0%)	0 (0%)	0 (0%)
alterada após a deglutição	0 (0%)	0 (0%)	0 (0%)	1 (2,6%)	1 (2,6%)	0 (0%)	1 (2,6%)	0 (0%)	0 (0%)	0 (0%)
										
**Voz molhada (clareamento laringeo espontâneo)**										
ausência	38 (100%)	36 (94,7%)	38 (100%)	37 (97,4%)	37 (97,4%)	37 (97,4%)	37 (97,4%)	35 (97,4%)	38 (100%)	37 (100%)
presença	0 (0%)	2 (5,3%)	0 (0%)	1 (2,6%)	1 (2,6%)	1 (2,6%)	1 (2,6%)	1 (2,6%)	0 (0%)	0 (0%)
										
**Voz molhada (clareamento laringeo voluntário)**										
ausência	38 (100%)	38 (100%)	38 (100%)	38 (100%)	37 (97,4%)	38 (100%)	37 (97,4%)	35 (97,4%)	38 (100%)	37 (100%)
presença	0 (0%)	0 (0%)	0 (0%)	0 (0%)	1 (2,6%)	0 (0%)	1 (2,6%)	1 (2,6%)	0 (0%)	0 (0%)
										
**Tosse**										
ausência	38 (100%)	36 (94,7%)	36 (94,7%)	36 (94,7%)	37 (97,4%)	38 (100%)	38 (100%)	36 (100%)	37 (97,4%)	37 (100%)
presença	0 (0%)	2 (5,3%)	2 (5,3%)	2 (5,3%)	1 (2,6%)	0 (0%)	0 (0%)	0 (0%)	1 (2,6%)	0 (0%)
										
**Tipo de tosse**										
forte	0 (0%)	2 (5,3%)	2 (5,3%)	2 (5,3%)	1 (2,6%)	0 (0%)	0 (0%)	0 (0%)	1 (2,6%)	0 (0%)
fraca	0 (0%)	0 (0%)	0 (0%)	0 (0%)	0 (0%)	0 (0%)	0 (0%)	0 (0%)	0 (0%)	0 (0%)
não se aplica	38 (100%)	36 (94,7%)	36 (94,7%)	36 (94,7%)	37 (97,4%)	38 (100%)	38 (100%)	36 (100%)	37 (97,4%)	37 (100%)
										
**Modo da tosse**										
reflexa	0 (0%)	1 (2,6%)	1 (2,6%)	1 (2,6%)	0 (0%)	0 (0%)	0 (0%)	0 (0%)	0 (0%)	0 (0%)
voluntária	0 (0%)	1 (2,6%)	1 (2,6%)	1 (2,6%)	1 (2,6%)	0 (0%)	0 (0%)	0 (0%)	1 (2,6%)	0 (0%)
não se aplica	38 (100%)	36 (94,7%)	36 (94,7%)	36 (94,7%)	37 (97,4%)	38 (100%)	38 (100%)	36 (100%)	37 (97,4%)	37 (100%)
										
**Momento da tosse**										
antes	0 (0%)	0 (0%)	0 (0%)	0 (0%)	0 (0%)	0 (0%)	0 (0%)	0 (0%)	38 (100%)	0 (0%)
durante	0 (0%)	2 (5,3%)	2 (5,3%)	2 (5,3%)	0 (0%)	0 (0%)	0 (0%)	0 (0%)	0 (0%)	0 (0%)
após	0 (0%)	0 (0%)	0 (0%)	0 (0%)	1 (2,6%)	0 (0%)	0 (0%)	0 (0%)	1 (2,6%)	0 (0%)
não se aplica	38 (100%)	36 (94,7%)	36 (94,7%)	36 (94,7%)	37 (97,4%)	38 (100%)	38 (100%)	36 (100%)	37 (97,4%)	37 (100%)
										
**Engasgo**										
ausência	38 (100%)	36 (94,7%)	38 (100%)	38 (100%)	38 (100%)	38 (100%)	38 (100%)	36 (100%)	38 (100%)	37 (100%)
presença: rápida recuperação	0 (0%)	2 (5,3%)	0 (0%)	0 (0%)	0 (0%)	0 (0%)	0 (0%)	0 (0%)	0 (0%)	0 (0%)
presença: recuperação com dificuldade	0 (0%)	0 (0%)	0 (0%)	0 (0%)	0 (0%)	0 (0%)	0 (0%)	0 (0%)	0 (0%)	0 (0%)
										
**Alteração de frequência cardíaca**										
ausência	38 (100%)	38 (100%)	38 (100%)	38 (100%)	37 (97,4%)	38 (100%)	38 (100%)	36 (100%)	37 (97,4%)	37 (100%)
presença	0 (0%)	0 (0%)	0 (0%)	0 (0%)	1 (2,6%)	0 (0%)	0 (0%)	0 (0%)	1 (2,6%)	0 (0%)
										
**Alteração de frequência respiratória**										
ausência	38 (100%)	37 (97,4%)	36 (94,7%)	36 (94,7%)	35 (92,1%)	38 (100%)	38 (100%)	36 (100%)	37 (97,4%)	36 (97,4%)
presença	0 (0%)	1 (2,6%)	2 (5,3%)	2 (5,3%)	3 (7,9%)	0 (0%)	0 (0%)	0 (0%)	1 (2,6%)	1 (2,6%)

* 2 missings;

** 1 missing

Os sintomas avaliados pela escala ESAS-r não apresentaram diferença estatística quando comparados por habilidade de deglutição, mas foi possível observar que pacientes com pior habilidade de deglutição apresentaram medianas maiores em todos os sintomas avaliados pela escala em comparação com aqueles com deglutição normal e também com as medianas da amostra total (Figura 1).

**Figura 1:** ESAS-r e deglutição

Por outro lado, quando se analisou as medianas dos sintomas avaliados por grau de funcionalidade, foi evidenciado que ´Dor´ apresentou diferença estatisticamente significante entre os grupos de funcionalidade (p=0,03), detalhado na Figura 2.

**Figura 2:** ESAS-r e PPS

O status nutricional encontrado se evidenciou pelos seguintes parâmetros: média 24kgm^2^ [±5,35] do IMC, ASG-PPP com média 6 [±3,63], caracterizando-se por moderadamente desnutridos, e as seguintes médias corporais e de força: circunferência de panturrilha direita 34,0cm [±5,52], circunferência de panturrilha esquerda 33,6cm [±5,32], força preensão palmar direita 20,0Kgf [±7,67] e força preensão palmar esquerda 20,2Kgf [±9,31].

Quanto aos sinais e sintomas associados à deglutição, os pacientes relataram alguns, à saber: Xerostomia (10%), Fadiga (15%), Alteração do paladar (8%). Além disso, ao aplicar o protocolo PARD, foram encontradas leves alterações em alguns parâmetros da dinâmica da deglutição, detalhados na Tabela 1.

Em relação às habilidades de deglutição, 74,4% apresentavam escore FOIS 7 - considerada o melhor padrão funcional da deglutição e 25,6% apresentavam escore FOIS 6 - apresentando leves alterações na habilidade de ingerir alimentos e líquidos. Não foram observados a presença de demais escores FOIS na presente amostra. Na classificação da deglutição proposta pelo protocolo PARD, os pacientes apresentaram em sua maioria deglutição normal (63,2%), enquanto 34,2% apresentam deglutição funcional, devido a presença de leves alterações que não impactavam na eficiência da deglutição e apenas um paciente apresentou disfagia orofaríngea leve (2,6%) devido a presença de tosse espontânea e pigarro voluntário eficazes, aliado a presença de leves alterações orais com compensações adequadas.

Finalmente se executou análise uni e multivariada da função de deglutição da amostra a fim de se evidenciar prováveis relações com o grau de funcionalidade e o estado nutricional, e ocorreram associações estatisticamente significantes entre os níveis da habilidade de deglutição e ECOG-PS; assim como com os resultados do protocolo nutricional ASG-PPP, conforme demonstrado na Tabela 2.

**Tabela 2:** Habilidade de deglutição segundo dimensões clínico-funcionais e nutricionais

MEDIDAS CATEGÓRICAS	FOIS 6 N=10	FOIS 7 N=29	*Odds Ratio* [95% IC]	valor-p
	N (%)			
**Sexo**				
Feminino	5 (18,5%)	22 (81,5%)	-----	0,394^RL^
Masculino	5 (41,7%)	7 (51,3%)		
**Xerostomia autorrelatada**				
Sim	2 (50,0%)	2 (50,0%)	-----	0,267^Q^
Não	8 (22,9%)	27 (77,1%)		
**Fadiga autorrelatada**				
Sim	1 (16,7%)	5 (83,3%)	-----	1,000^Q^
Não	9 (27,3%)	24 (72,7%)		
**Disgeusia autorrelatada**				
Sim	1 (33,3%)	2 (66,7%)	-----	1,000^Q^
Não	9 (25,0%)	27 (75,0%)		
**Pneumonia recente autorrelatada**				
Sim	2 (66,7%)	1 (33,3%)	-----	0,256^RL^
Não	8 (22,2%)	28 (77,8%)		
**Status Nutricional**				
Bem nutrido	1 (6,7%)	14 (93,3%)	-----	0,331RL
Moderadamente à Gravemente desnutrido**	9 (37,5%)	15 (62,5%)		
**PPS**				
PPS < OU = 60	8 (34,8%)	15 (65,2%)	-----	0,126^RL^
PPS > OU = 70	2 (12,5%)	14 (87,5%)		
MEDIDAS CONTÍNUAS	(Média/Mediana[Desvio Padrão])		*Hazard Ratio* [95% IC]	
CCEB (escore total)	31,50 [±8,90]	32,00 [±9,71]	-----	0,856^RL^
Idade (anos)	73 [±17,26]	69 [±17,04]	-----	0,518^RL^
IMC (Kg/m2)	25,84 [±6,26]	24,63 [±5,29]		0,489^M^
*Handgrip* D (kgf)+	19 [±7,79]	20 [±7,52]	-----	0,488^M^
*Handgrip* E (kgf)^+^	19 [±10,31]	21 [±9,70]	-----	0,459^M^
Circunferência de Panturrilha D (cm)^++^	34 [±5,52]	34 [±4,62]	-----	0,390^M^
Circunferência de Panturrilha E (cm)++	34 [±6,46]	34 [±4,71]	-----	0,298^M^
ASG-PPP	8 [±5,42]	5 [±3,01]	0,76 [0,59 - 0,98]**	0,040^RL^
ECOG-PS	2,17 [±0,75]	1,25 [±0,85]	0,23 [0,57 - 0,95]**	0,044^RL^
PPI	2,83 [±1,57]	1,28 [±1,90]	-----	0,367^RL^

Q Qui-quadrado;

M Mann-Whitney;

** OR ajustado segundo análise multivariada de FOIS ajustada por idade, sexo, status nutricional, pneumonia recente, PPS e PPI (Backward p<0,25);

RL valor-p segundo o respectivo último modelo de Regressão Logística;

+ 5 missings;

++ 8 missings

## DISCUSSÃO

Nesse estudo, em que se avaliou a função de deglutição segundo o grau de funcionalidade e o estado nutricional de pacientes em cuidados paliativos oncológicos, exceto aqueles que abarcavam regiões anatômicas da deglutição, encontrou-se como principais resultados associações entre mínima mudança na habilidade de deglutição e piores níveis tanto da funcionalidade global, quanto nutricional desta amostra. Ainda que não sabida a raiz dessa causalidade, são dados a serem considerados.

A população estudada apresentou 25,6% da amostra com leves alterações na habilidade de ingerir alimentos e líquidos segundo FOIS e devido a presença de compensações espontâneas e eficazes nas fases oral e faríngea da deglutição, sendo que toda nutrição e hidratação se mantinham por via oral. Corroborando com os achados de outro estudo transversal de pacientes com câncer fora das regiões anatômicas de deglutição, que considerou presença de disfagia se escala FOIS menor que 7, no qual a disfagia ocorreu em 19%, sendo que naqueles que estavam em CP, a prevalência foi de 30%. Deste modo, ambos estudos destacam uma nova forma de considerar a funcionalidade da deglutição, além da importância de se excluir os tipos de câncer de cabeça, pescoço e trato gastrointestinal superior de tais análises.

Por outro lado, resultados semelhantes a estes foram observados em pesquisas com pacientes com qualquer tipo de câncer avançado incluindo áreas anatômicas da deglutição. Em outro estudo transversal, realizado em cenário ambulatorial, com pacientes com qualquer tipo de câncer, observaram as seguintes habilidades de deglutição segundo a escala FOIS: 56,7% escore 7 e 23,2% escore 6^(32)^. Uma coorte prospectiva italiana^(33)^ observou disfagia em 15% do total de pacientes encontrados, sendo que destes, a maioria foi classificada como transtorno de deglutição que afetava parcialmente a nutrição dos indivíduos, sem necessidade de suplementação oral ou via alternativa. Estes resultados destacam a importância de distinguir etiologicamente as alterações anatomofisiológicas que impactam a função de deglutição. Nos casos de câncer de cabeça e pescoço, incluindo casos avançados, essas modificações ocorrem devido às deformações estruturais musculares, ósseas e cartilaginosas do trato digestório e/ou respiratório^(34)^. Nos casos de câncer do sistema nervoso central, as modificações estruturais neurológicas causam disfagias neurogênicas^(11)^. À vista disso, as disfagias resultantes de doenças oncológicas fora das áreas anatômicas da deglutição parecem ser explicadas pela degradação clínica e funcional promovida pelo câncer e seus tratamentos, principalmente quimioterapia^(7,11)^. O impacto na perda de força e mobilidade da musculatura corporal global durante o tratamento oncológico inclui os músculos envolvidos na deglutição^(11)^. Esse processo parece afetar mecanismos biomecânicos e sensoriais da deglutição - como visto na presente amostra, possivelmente contribuindo para o surgimento de disfagia orofaríngea ou esofágica por decréscimo da funcionalidade^(11)^, o que propomos denominar de disfagia por declínio funcional - um sintoma do tipo gastrointestinal que necessita ser manejado principalmente em pacientes com câncer avançado.

A relação entre habilidade de deglutição prejudicada, mesmo que discreta, e pior grau de funcionalidade na amostra do presente estudo também corrobora com o impacto musculo-funcional que o universo oncológico proporciona na habilidade de deglutição mencionado por Okuni et al.^(11)^. Ademais, pesquisadores italianos^(33)^ também encontraram relação da disfagia como um baixo nível em escala de funcionalidade. Em outros dois estudos em pacientes com câncer avançado^(10,35)^ os pesquisadores também descobriram a disfagia como um dos sintomas mais comuns nos últimos sete dias de vida e últimas horas de vida, respectivamente, o que mostra alta degradação clínico-funcional relacionada a este sintoma gastrointestinal.

A relação de declínio funcional com alguns sintomas da escala ESAS-r encontrado neste estudo corrobora com achados de outros pesquisadores brasileiros^(36)^, que também em um cenário ambulatorial paliativo, encontraram ‘dor’ (média e mediana de 4,04 e 5,0, respectivamente), e ‘ansiedade’ (3,85 e 4,0) como os sintomas mais prevalentes, entretanto eles não realizaram análise de relação com status funcional.

Quanto aos aspectos nutricionais, a presente amostra evidenciou relação estatisticamente significante com pior funcionalidade e também com a presença de alterações na habilidade de deglutição. A relação com a funcionalidade também foi encontrada por Oliveira et al.^(37)^, em uma população de brasileiros com diversos tipos de câncer incuráveis atendidos em sua maioria em clínicas oncológicas, nos quais as melhores qualificações no protocolo ASG-PPP refletiam em melhores aspectos físicos e de qualidade de vida geral. No entanto, outro estudo transversal brasileiro, com pacientes oncológicos em assistência paliativa exclusiva, apresentou ausência de associações entre performance funcional segundo PPS e aspectos nutricionais avaliados, sendo que essa falta de associação pode estar relacionada ao tamanho da amostra reduzido, justificativa evidenciada inclusive pelos próprios autores. Já a relação do estado nutricional com a disfagia, a literatura nacional^(38,39)^ e internacional^(4,33,40)^ há algum tempo ressalta tal associação, e os achados do presente estudo estão em consonância a comunidade cientifica.

Em vista do crescente número de pacientes com câncer avançado em todo o mundo, destaca-se a necessidade de fortalecer as políticas públicas voltadas para o modelo de assistência paliativa, visando a composição da equipe de cuidados paliativos com profissional de diferentes áreas, em todos os níveis de atenção à saúde sendo cenários públicos ou privados. Além disso, torna-se imperativo que equipes de saúde conheçam as múltiplas necessidades de cuidado que permeiam a complexidade e singularidade que pacientes com câncer avançado exige^(41)^, sendo que as alterações das funções orais compõem essa complexa deterioração clínico-funcional, inclusive em regiões que não abarcam cabeça, pescoço e trato gastrointestinal superior e por isso precisam ser mais bem reconhecidas e manejadas^(42)^. Destaca-se aqui o papel crucial da deglutição para a nutrição, prazer e qualidade de vida desses pacientes.

Existem algumas limitações em nosso estudo. Primeiro, não foi utilizada avaliação instrumental, como a videofluoroscopia da deglutição (VFD), devido à logística geográfica desfavorável, já que seria necessário o deslocamento dos pacientes até o local de realização do exame, visto que o local de coleta não dispunha do equipamento. Em contraponto, a escolha do protocolo que avalia mais de uma consistência alimentar, como o PARD, se deu justamente por este tipo de avaliação apresentar sensibilidade de 90%, em comparação com VFD^(43)^, evidenciando a equivalência da avaliação clínica completa, que pode ser considerada soberana. Segundo, este estudo possivelmente não controlou todos os possíveis fatores de confusão, derivados de uma amostra tão heterogênea. No entanto, realizamos análises multivariadas o que conferiu maior robustez aos resultados. Em terceiro lugar, o viés de seleção dos participantes deve ser considerado. A amostra foi constituída por conveniência devido à dificuldade de recrutamento, sendo que a condição de saúde deteriorada foi um dos motivos de recusa. Essas barreiras relacionadas ao paciente acompanhado por equipe de cuidados paliativos também foram identificadas por outros autores^(44,45)^.

Apesar dessas limitações, um ponto forte crucial é que esta pesquisa fornece a prevalência da disfagia em pacientes com câncer que não abarcam áreas anatômicas da deglutição e sua relação com a degradação clínico-funcional e nutricional por meio de desfecho primário, o que credibiliza nossos achados e muito contribuem para melhorar a assistência a esses pacientes e para a área de CP.

Ressalta-se a importância de realizar mais pesquisas randomizadas, incluindo estudos multicêntricos, para aprofundar a compreensão dos sintomas e seus impactos, além de fazer uma análise aprofundada dos resultados da abordagem holística em pacientes com doenças sem possibilidade de cura e seus familiares.

## CONCLUSÃO

Em conclusão, este estudo evidencia que a habilidade de deglutição prejudicada, mesmo sendo discreta nesta amostra de pacientes com câncer avançado que não abarcam a região de cabeça, pescoço e trato gastrointestinal superior, apresenta relação com grau de funcionalidade inferior e estado nutricional deficiente.

## Material Suplementar

Este artigo acompanha material suplementar.

Projeto piloto e análise de concordância entre examinador

Este material está disponível como parte da versão online do artigo na página https://doi.org/10.1590/2317-1782/e20240210pt
